# Mixed reality‐based technology to visualize and facilitate treatment planning of impacted teeth: Proof of concept

**DOI:** 10.1111/ocr.12803

**Published:** 2024-05-07

**Authors:** Piotr S. Fudalej, Agnieszka Garlicka, Damian Dołęga‐Dołegowski, Magda Dołęga‐Dołegowska, Klaudia Proniewska, Iva Voborna, Ivana Dubovska

**Affiliations:** ^1^ Department of Orthodontics Jagiellonian University Medical College, Jagiellonian University in Cracow Krakow Poland; ^2^ Department of Orthodontics and Dentofacial Orthopedics, Medical Faculty, School of Dental Medicine University of Bern Bern Switzerland; ^3^ Faculty of Medicine and Dentistry, Institute of Dentistry and Oral Sciences Palacký University Olomouc Olomouc Czech Republic; ^4^ Jagiellonian University Medical College Jagiellonian University in Cracow Krakow Poland

**Keywords:** AR, VR, HoloLens 2 goggles, impacted canine, mixed virtual reality

## Abstract

**Objective:**

We propose a method utilizing mixed reality (MR) goggles (HoloLens 2, Microsoft) to facilitate impacted canine alignment, as planning the traction direction and force delivery could benefit from 3D data visualization using mixed reality (MR).

**Methods:**

Cone‐beam CT scans featuring isometric resolution and low noise‐to‐signal ratio were semi‐automatically segmented in Inobitec software. The exported 3D mesh (OBJ file) was then optimized for the HoloLens 2. Using the Unreal Engine environment, we developed an application for the HoloLens 2, implementing HoloLens SDK and UX Tools. Adjustable pointers were added for planning attachment placement, traction direction, and point of force application. The visualization was presented to participants of a course on impacted teeth treatment, followed by a 10‐question survey addressing potential advantages (5‐point scale: 1 = totally agree, 5 = totally disagree).

**Results:**

Out of 38 respondents, 44.7% were orthodontists, 34.2% dentists, 15.8% dental students, and 5.3% dental technicians. Most respondents (44.7%) were between 35 and 44 years old, and only 1 (2.6%) respondent was 55–64 years old. Median answers for six questions were ‘totally agree’ (25th percentile 1, 75th percentile 2) and for four questions ‘agree’ (25th percentile 1, 75th percentile 2). No correlation was found between age, profession, and responses.

**Conclusion:**

Our method generated substantial interest among clinicians. The initial responses affirm the potential benefits, supporting the continued exploration of MR‐based techniques for the treatment of impacted teeth. However, the recommendation for widespread use awaits validation through clinical trials.

## 1 INTRODUCTION

Impacted and displaced teeth are relatively common in humans. These are teeth that are unable to erupt naturally because they are obstructed by other teeth or bone (impacted), or teeth that have an abnormal position (displaced). Depending on the population being studied, the prevalence of impaction and displacement can range from 1 to 7.5%.^1^, ^2^, ^3^

Although any tooth can be impacted or displaced, impaction of the maxillary canines is among the most common after third molars and can lead to various complications. Root resorption of adjacent teeth, typically the maxillary lateral incisors, and sometimes even the central incisors and premolars, is a potential complication that can necessitate the removal of adjacent teeth.^4^ The use of advanced imaging techniques, such as cone‐beam computed tomography (CBCT), is allowing more frequent detection of root resorption. For example, Walker et al.^5^ found root resorption in 67% of patients, 11% of which were central incisors and 4% premolars. Similarly, Alqerban et al.^6^ detected root resorption in 54% of lateral incisors and 15% of central incisors.

The success of treatment of an impacted tooth depends on aligning the tooth into the correct position in the dental arch without causing damage to other teeth. However, this can be particularly difficult when the roots of the impacted canine and adjacent teeth are intertwined, such as in cases of partial transposition, or when the crown of the impacted canine is in direct contact with an adjacent root or the direction of traction must be changed during alignment. For example, Chaushu et al.^7^ described a so‐called labial‐palatal impacted canine. Its unusual position is often misdiagnosed, leading to alignment failure. In general, mistaken positional diagnosis and erroneous traction direction have been reported as the second most common factor responsible for failure to resolve maxillary canine impaction.^8^ However, planning the traction direction can be challenging, particularly when collaboration between the orthodontist, dental surgeon, and laboratory is required to establish the exact placement of the attachment to the impacted tooth and/or design an appliance that generates the appropriate orthodontic force. In such scenarios, effective communication through technology that visualizes the region of interest and facilitates exchange of information among all parties involved in the therapeutic process is indispensable.

One of the challenges in presenting CBCT data on a flat screen is that 3D data is presented in 2D, resulting in several limitations, including loss of depth perception, distortion, and complexity.^9^ Although volume rendering is frequently used to address these issues, some limitations persist.^10^ Manipulating a CBCT scan in 3D on a flat screen provides some advantages, such as the ability to zoom in and out, rotate and pan the image, and adjust the opacity of the different structures. However, presenting a CBCT scan in a true 3D environment could provide additional advantages, such as improved depth perception, enhanced visualization through a more immersive experience, and interactivity.

**FIGURE 1:**
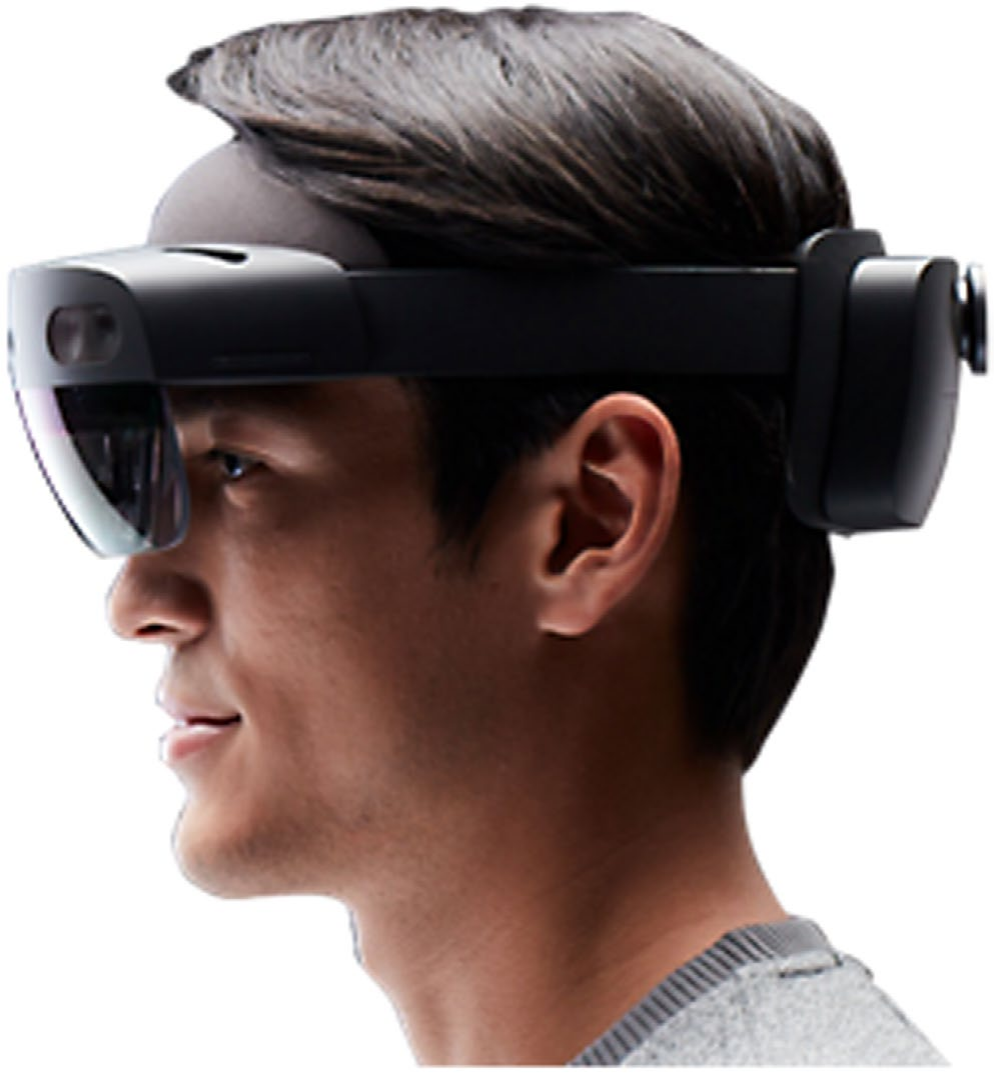
HoloLens 2 goggles (from: https://www.microsoft.com/en‐us/hololens).

Among the various techniques for presenting 3D data as a 3D object, mixed reality (MR) is promising, as it allows for a genuine 3D visualization of data. MR glasses (Figure 1) provide an immersive experience that allows users to interact with virtual objects as if they were in the real world, enabling users to view and interact with them more naturally. Other methods, such as 3D printing,^11^ also exist but are not as promising for true 3D visualization of impacted teeth.

**FIGURE 2:**
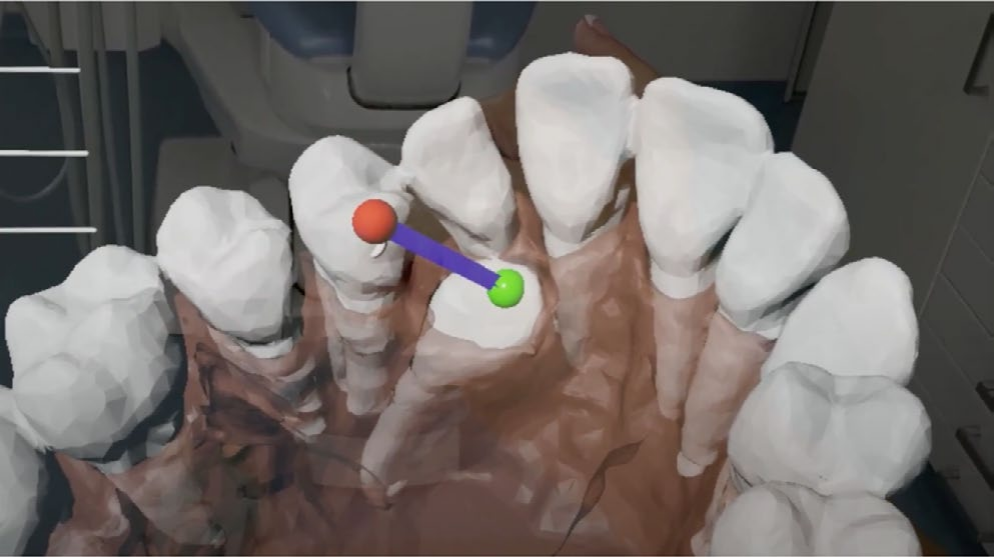
Visualization of the upper right impacted canine by HoloLens 2 goggles. The green sphere denotes the position of the orthodontic attachment (e.g., button with a gold chain) on the impacted canine crown. The red sphere is positioned to optimize the traction direction (line connecting both spheres).

**FIGURE 3:**
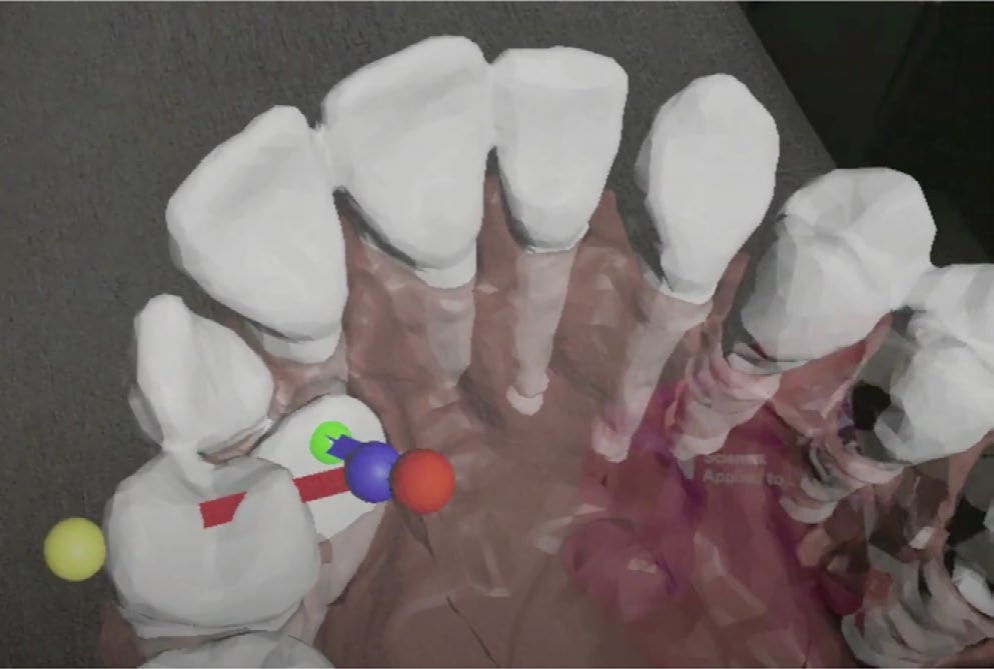
Visualization of the upper right impacted canine by HoloLens 2 goggles. The violet line represents the initial traction direction for uprighting and canine extrusion. The red line indicates the second traction direction towards the dental arch. The cross‐section where the lines intersect marks the point where the traction direction needs to be changed. Teeth 14 and 24 were extracted before active traction due to a class II malocclusion.

MR also facilitates communication between different clinicians and with a patient. This enables collaborative planning with a laboratory when designing an appliance for traction of the impacted tooth, providing clear instructions to the dental surgeon on where to attach the button for a chain used for traction, or explaining the chosen treatment method to the patient in a more understandable way (Figures 2 and 3).

The purpose of this article is to propose a method using HoloLens 2 goggles equipped with MR technology to facilitate planning of orthodontic traction for the alignment of impacted teeth and communication among all parties involved in treatment of impactions, including the orthodontist, dental surgeon, technician, and, most importantly, the patient.

## 2 MATERIALS AND METHODS

The effectiveness of and patient satisfaction with the method described below is currently being tested in a two‐centre randomized prospective clinical trial. The study was approved by the bioethics committees of the Jagiellonian University in Krakow (Poland) and Palacký University in Olomouc (Czech Republic) (1072.6120.130.2021 and 16/22). All steps related to the study, including this submission, were performed on anonymized material while respecting the Declaration of Helsinki and the principles of Good Clinical Practice (GCP). Study participants provided written consent to participate in the investigation.

According to study protocol, each participant had a CBCT scan prior to exposure of the impacted canine and placement of the attachment for traction. Depending on the group assignment. HoloLens (+) or HoloLens (−), visualization of the position of the impacted canine relative to adjacent teeth and bones was or was not prepared for HoloLens 2 goggles, respectively, for planning the traction direction and manufacturing an appliance with which to align the canine. For the current report, we randomly chose a patient from the HoloLens (+) group, that is, for whom a holographic visualization was prepared.

### 2.1 Image acquisition, data processing, and segmentation

CBCT scans were performed using a Cranex 3Dx machine (Soredex Oy, Tuusula, Finland) with a field‐of‐view of 5 × 5 cm and a resolution of 0.2 mm (voxel size). The resulting data set was exported using the Digital Imaging and Communications in Medicine (DICOM) file format.

The CBCT DICOM file was processed using Inobitec DICOM Viewer Pro software (v.2021, Inobitec LLC, Voronezh, Russia) to segment the maxilla, impacted canine, and adjacent teeth. The software was used to import the file, which had isometric resolution and a low noise‐to‐signal ratio. The segmentation process involved several steps.

First, the maxilla, including all teeth, was segmented using the ‘region growing’ tool with a pre‐selected density threshold range expressed in grey values. The ‘cut’ tool was then used to manually remove any artefacts. Next, the impacted canine and adjacent teeth were segmented by identifying and selecting them on one of the CBCT slices using the ‘select’ tool. The software automatically identified the teeth on other slices once they were identified on one slice. The ‘cut’ tool was used again to remove any artefacts.

The segmented teeth were then duplicated and enlarged by one voxel, subtracted from the entire maxilla, and added back to the maxilla as duplicates. The volumetric image of the maxilla and teeth was converted into a 3D mesh (OBJ file) in which artefacts could be easily removed using Blender tools. Finally, the mesh resolution was optimized for the HoloLens 2 device by reducing the number of triangles in the mesh to approximately 100 000–150 000 for the maxilla and 40 000 for the tooth.

### 2.2 Preparation of the MR application

To create a MR application using the HoloLens 2 device, we needed a 3D model and application created in the Unreal Engine with appropriate setup in the way that it understands hand gestures (e.g., scaling, moving, and rotating) and moves with direction glasses.

When the application was set, we added it to the 3D model and then created pointers to mark appropriate points on the maxilla and tooth by adding four spheres, each with a different colour and functionality that allowed them to be moved around in space. A function was also written to draw a line between spheres 1 and 2, and spheres 3 and 4.

The same application can be used for each case covered by the study. The imported mesh needs to be replaced with a new one and set up with a new unique ID for each case. When the application is used for the first time, the user can move and set the position of the spheres (pointers) in space (Figures 2 and 3) and then read it as coordinates (X, Y, Z), which are later written to the server in a JSON file. The next time the application is started, the pointers will be set in the earlier positions.

### 2.3 Assessing acceptance of the method

Participants in the clinical course on the management of impacted canines, including orthodontists, general dentists, dental students, and technicians, were introduced to the functionality of the application and its features. Initially, there was an explanation of MR technology, along with a discussion of the potential advantages and limitations in the management of impacted teeth. Following this, each participant was presented with HoloLens 2 goggles featuring an illustrative visualization of severe impaction of a maxillary canine on one side. Subsequently, the session covered a method for determining the placement of attachments, the direction of traction, and the design of orthodontic appliances used for aligning impacted canines. Participants were encouraged to pose questions throughout the session. Finally, participants were provided a 14‐item questionnaire comprising 2 demographic questions (profession and age), 2 open‐ended questions (identifying the three main advantages and disadvantages of the method), and 10 questions constructed based on the opening, ‘Do you agree that visualization of impacted canines with the HoloLens 2 goggles allows for…’ (Table 1). Responses were collected on a 5‐point scale (1 = totally agree, 5 = totally disagree). Answers of ‘I do not know’ were allowed and recorded.

**TABLE 1:** Demographics and descriptive statistics of responses to questions.

Question	*N*	Mean	SD	Median	25th %	75th %	Min	Max
Age category	38	N/A	N/A	3	2	3	1	6
Do you agree that visualization of impacted canines with the HoloLens 2 goggles allows for:								
Precise planning of the placement of the orthodontic attachment?	36^a^	1.5	0.7	1	1	2	1	3
Precise planning of the surgical procedure?	35	1.5	0.7	1	1	2	1	3
A reduced need for an orthodontist to be present in the surgery room?	35	1.9	0.9	2	1	2	1	5
Thorough planning of the direction of the active traction?	35	1.5	0.6	1	1	2	1	3
Precise planning of the appliance for active traction?	33	1.6	0.7	1	1	2	1	3
A reduction of adverse effects?	35	1.7	0.7	2	1	2	1	3
A positive effect on the outcome of impacted canine treatment?	37	1.8	0.8	2	1	2	1	4
A reduction of treatment time?	34	2.0	0.9	2	1	3	1	4
Enhancement of the patient's/parent's education and communication with them?	37	1.6	0.8	1	1	2	1	4
Improvement in dental student education?	36	1.6	0.7	1	1	2	1	3

^a^ Responses of ‘I do not know’ were not included in the statistical analysis.

### 2.4 Statistical analysis

Respondents were grouped according to age into six categories: <25, 25–34, 35–44, 45–54, 55–64, and >65 years old. Responses of ‘I do not know’ to survey questions were not included in the statistical analysis.

Descriptive statistics included calculation of mean, standard deviation, median, 25th and 75th percentile, and range for individual answers. Correlation analysis was carried out to identify associations between profession, age group, and individual answers. PAST v.4 software (Natural History Museum – University of Oslo, Oslo, Norway) was used for statistical analyses.

## 3 RESULTS

Thirty‐eight participants responded to the questionnaire: 44.7% were orthodontists, 34.2% were general dentists, 15.8% were dental students, and 5.3% were technicians. Most respondents (44.7%) were between 35 and 44 years old; 1 (2.6%) respondent was in the 55–64 age group. Median answers for six questions were ‘totally agree’ (25th percentile 1, 75th percentile 2), whereas four questions received a median response of ‘agree’ (25th percentile 1, 75th percentile 2; Table 1).

Respondents listed better visualization of the impacted canine and improved precision to plan orthodontic traction as being among the main advantages, whereas the price of the HoloLens 2 goggles and the need to involve a technician to segment the CBCT scan were among the main disadvantages of the proposed visualization method.

No significant correlation was found between profession or age and responses.

## 4 DISCUSSION

The rapid development and widespread use of new technologies has significantly impacted various aspects of our daily lives. Dentistry is no exception to this phenomenon, as various novel technologies are being utilized in different dental procedures.^12^

Our proposed solution involves the use of HoloLens 2 goggles with a customized application that provides a visualization of the impacted canine area, which can enhance communication among the orthodontic treatment team for patients with impactions. By utilizing a graphic representation of the direction of the orthodontic traction, the incidence of complications, particularly in complex cases, can be minimized (Figure 3). Visualization of orthodontic traction helps the orthodontist direct traction more accurately and change the direction of traction at a precise point, which speeds up treatment and increases its effectiveness. This also helps the technician produce an accurate 3D‐printed transpalatal bar with arms in which the eyelets are in the right place to direct traction.

Furthermore, indicating the appropriate attachment bonding site for the impacted canine can facilitate tooth exposure and attachment bonding (Figure 2), avoiding the complications caused by incorrect attachment placement.^7^ The proposed solution also has the advantage of visualizing the impacted canine and its potential movement into the dental arch for the patient. Three‐dimensional observation may facilitate patient acceptance of the proposed orthodontic appliance and reduce the stress associated with the surgical procedure.

In the field of dentistry, MR has been applied in educational training in university settings, surgical treatment/implantology, and treatment of patients with dental phobia.^13^, ^14^ A recent systematic review showed promising outcomes of MR technologies in dental education and implant placement. Moussa et al.^15^ concluded that virtual technology enhances the education outcomes of dental students. Mai et al.^16^ reported that AR‐navigated implant placement had clinically acceptable accuracy comparable to implant‐guided surgery. Yan et al.^17^ demonstrated the potential of MR distraction interventions in alleviating dental anxiety among paediatric patients. However, no clinical orthodontic applications of MR technology have been reported to date.

Modern medicine places the patient at the centre of care, where the patient must be thoroughly informed about their condition, treatment options, and associated risks. Based on the information provided by the healthcare professional, the patient gives informed consent, which is a voluntary agreement to undergo the proposed treatment. In the context of MR technology in dentistry, any visualization method that provides supplementary information to the patient or presents it in a more comprehensible way could improve effective communication. The purpose of such methods, including the proposed solution, would be two‐fold, to facilitate patients' acceptance of treatment and to reduce fear associated with treatment, particularly surgical procedures. Although the proposed method has not yet been clinically tested, its potential appears to be significant.

**FIGURE 4:**
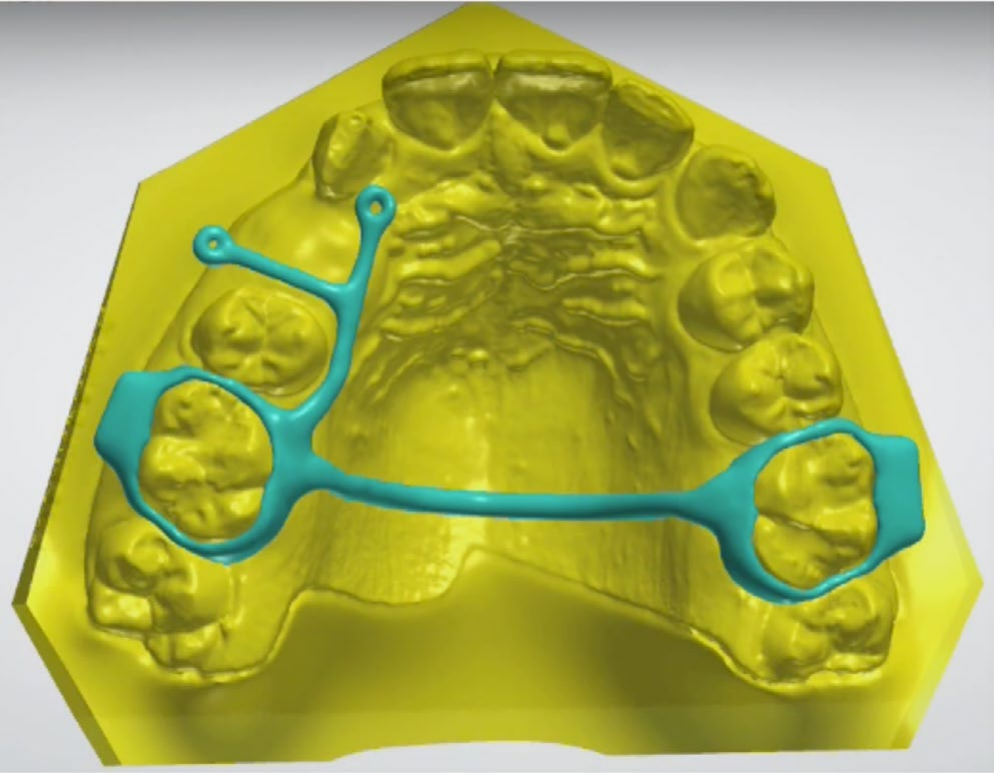
Visualization of a 3D‐printed transpalatal bar. Eyelets for active traction were placed based on the visualization using HoloLens 2 goggles. Tooth 14 was virtually extracted according to planned extractions of teeth 14 and 24 in the treatment plan.

Digital dentistry and digital workflow are terms that imply modern and efficient dentistry. The digitalization of procedures, or the entire workflow, is expected to make the treatment process easier, improve organization, speed up communication with the laboratory, and provide the patient with information about their treatment. All of this should be done while maintaining cost‐effective treatment. Bernauer et al.^18^ reported that, in fixed prosthodontics, a complete digital protocol, which involves using an intraoral scanner to three‐dimensionally capture the situation in the patient's mouth, designing and manufacturing the restoration in a fully virtual environment, and clinically delivering the dental restoration, is comparable to traditional workflows in terms of time efficiency, production costs, precision, and patient satisfaction. Although a complete digital workflow is not yet available in orthodontics, the current solution could still be a valuable addition to the treatment of severely impacted teeth. The 3D visualization capabilities of the technology can be utilized for communication and appliance design (Figure 4), making it a promising tool for orthodontic practitioners.

The HoloLens 2 system, while innovative, has its limitations. First, the price of the equipment can be a significant barrier, with the cost of the HoloLens 2 starting at more than $3500. Second, the procedure is not fully automatic, and manual segmentation and the creation of visualization applications can be laborious and not cost‐effective. Finally, though visualization of the impacted canine area is impressive, it cannot be superimposed onto the patient's real situation, which could potentially facilitate surgery. However, it is likely that advancements in technology will overcome the current limitations of MR systems and make them more accessible and affordable for a wider range of users. As computing power continues to increase and the cost of components decrease, MR systems may become more lightweight, portable, and capable of delivering higher‐quality visualizations. Furthermore, as the demand for MR technology grows, the market will become more competitive, leading to reduced costs.

## 5 CONCLUSIONS

Our method has generated substantial interest among clinicians. The initial responses affirm the potential benefits, supporting the continued exploration of AR‐based techniques in the treatment of impacted teeth. However, the recommendation for widespread use awaits validation through clinical trials.

## AUTHOR CONTRIBUTIONS

Conceptualization: P.S.F. and I.D.; methodology: P.S.F.; software: D.D.D.; validation: I.D., D.D.D., and M.D.D.; formal analysis: P.S.F.; investigation: I.D.; resources: I.V.; data curation: A.G.; writing—original draft preparation: P.S.F.; writing—review and editing: I.D.; visualization: M.D.D and D.D.D.; supervision: P.S.F.; project administration: K.P.; and funding acquisition: K.P. All authors have read and agreed to the published version of the manuscript.

## FUNDING INFORMATION

This study was partially supported by grant Erasmus+ No. 2020‐1‐PL01‐KA203‐HE‐082077.

## ETHICS STATEMENT

The Bioethics Committees of Jagiellonian University in Kraków, Poland, and Palacky University Olomouc, Olomouc, Czech Republic, have approved the study (1072.6120.130.2021 and 16/22, respectively). All steps related to the study, including the current submission, were performed on anonymized material, respecting the Declaration of Helsinki and the principles of good clinical practice (GCP).

## Data Availability

Data available on request from the authors.
